# Need for prolonged immunosupressive therapy in CLIPPERS-a case report

**DOI:** 10.1186/1471-2377-13-49

**Published:** 2013-05-24

**Authors:** Juerd Wijntjes, Ernest J Wouda, Carl EH Siegert, Giorgos B Karas, Annemarie MM Vlaar

**Affiliations:** 1 Department of Neurology, Sint Lucas Andreas Hospital, Jan Tooropstraat 164, 1061 AE, Amsterdam, Netherlands; 2 Department of Internal Medicine, Sint Lucas Andreas Hospital, Amsterdam, Netherlands; 3 Department of Radiology, Sint Lucas Andreas Hospital, Amsterdam, Netherlands

**Keywords:** Chronic lymphocytic inflammation with pontine perivascular enhancement responsive to steroids, CLIPPERS, Ataxia, Diplopia

## Abstract

**Background:**

Chronic lymphocytic inflammation with pontine perivascular enhancement responsive to steroids (CLIPPERS) was first described in 2010 by Pittock and colleagues. All reported patients presented with diplopia and gait ataxia and had similar typical MRI findings with punctuate gadolinium enhancement of the pons. Alternative diagnoses were excluded by means of laboratory, radiological and histological tests. All patients were successfully treated with steroids. We present a case in which the steroid therapy was switched to long term immunosuppressive therapy, leading to several severe side-effects, but sustained clinical improvement.

**Case presentation:**

A 63-year-old male presented with sub-acute diplopia and progressive gait ataxia. During admission his neurological condition worsened and he developed multiple cranial nerve deficits, paraparesis and urine retention. MRI-findings were remarkable with punctuate enhancement with gadolinium of the pons. Cerebrospinal fluid only showed elevated protein levels and all other additional investigations were normal. The probable diagnosis of CLIPPERS was made and intravenous corticosteroids were administered. This led to rapid clinical recovery and decreased enhancement on the MRI-scan. Long-term oral immunosuppressive therapy was started. One-and-a-half year later our patient has no recurrence of neurological symptoms, however due to the side effects of the immunosuppressive therapy he was readmitted several times.

**Conclusion:**

CLIPPERS presents with distinctive clinical and MRI-findings and may be diagnosed after excluding other differential diagnoses. Patients are treated with corticosteroids with good clinical results. Since short term glucocorticoid treatment results into relapse of the disease, longer term immunosuppressive therapy appears to be mandatory for sustained improvement, although accompanied by severe side effects.

## Background

Chronic lymphocytic inflammation with pontine perivascular enhancement responsive to steroids (CLIPPERS) was first described in 2010 by Pittock et al. [1] They described eight patients presenting with similar clinical and MRI-findings: sub-acute diplopia, gait ataxia and punctiform gadolinium enhancement “peppering” the pons. As distance from the pons increased, lesions became less numerous. Other accompanying clinical symptoms included dysartria, dysesthesia of the face and finally paraparesis and urine retention. The main manifestations of CLIPPERS are summarized in Table 1.

**Table 1:** **Core features of CLIPPERS (adapted from Simon et al.)**[2]

**Clinical:**	
**-**	subacute progressive ataxia and diplopia
**Radiological:**	
**-**	numerous punctate or nodular enhancing lesions bilaterally within one of the three following anatomical locations: pons, brachium pontis (=middle cerebellar punducle), cerebellum
**-**	individual radiological lesions are small but may coalesce to form larger lesions
**-**	lesions may occur in the spinal cord, basal ganglia or cerebral white matter but should be decreasing density with increasing distance from the pons.
**Corticosteroid responsiveness:**	
**-**	prompt and significant clinical and radiological response to corticosteroids
**Histopathological:**	
**-**	white matter perivascular lymphohistiocytic infiltrate with or without parenchymal extension.
**-**	infiltrate contains predominantly CD3 + and CD20+ lymphocytes.
**-**	absence of the following histopathological characteristics:
	○ monoclonal or atypical lymphocyte population
	○ necrotising granulomatomas or giant cells
	○ histological features of vasculitis
**Differential diagnosis should be excluded:**	
**-**	CNS lymphoma, glioma, primary CNS vasculitis, paraneoplastic syndrome, sarcoidosis, demyelinating disease, Behcet’s and Sjogren disease, tuberculosis, neurolues, Whipple’s disease and histiocytosis.

Since 2010, several new possible cases have been published (Table 2). Despite extensive diagnostic work-up so far, the pathogenesis had not been elucidated. An immune-mediated process has been postulated on the basis of the establishment of T-cell predominant infiltrates of the affected brain lesions and radiologic resolution of the lesions upon immunosuppressive treatment.

**Table 2:** Summary of published CLIPPERS case reports; initial- and follow up therapy

**Author**	**Cases**	**Biopsy**	**Initial treatment**	**Maintenance treatment**	**Follow up (months)**	**Therapy stopped**	Decline***
Pittock et al. [1]	8	4	prednisone	1 patient: prednisone and methotrexate	144	no	6/8
				1 patient: prednisone	25 (died)	no	
				1 patient: prednisone and azathioprine	43	no	
				1 patient: prednisone and mitoxantrone	14	no	
				4 patients: prednisone	20, 7, 22 and 7	no	
Simon et al. [2]	5	5	prednisone	1 patient: prednisone and methotrexate	12	no	5/5
				2 patients: prednisone, azathioprine and cylophosphamide	36, 72	no	
				1 patient: prednisone, mycophenolate and cylophosphamide	64	no	
				1 patient: prednisone and cylophosphamide	100	no	
Taieb et al. [3]	1	1	prednisone	prednisone and rituximab	9	no	unknown
Jones et al. [4]	2*	1*	prednisone	prednisone	> 6	no	1/1
Sempere et al. [5]	1	-	prednisone	prednisone and methotrexate	6	no	unknown
Duprez et al. [6]	1	-	prednisone	unknown	> 3	unknown	unknown
List et al. [7]	1	-	prednisone	prednisone and methotrexate	> 3	no	unknown
Limousin et al. [8]	1**	1**	-	-	-	-	-
Gabilondo et al. [9]	1	-	prednisone	prednisone, immunoglobulins iv once and azathioprine	9	no	1/1
Kastrup et al. [10]	3	3	2/3 prednisone	1 patient: prednisone and cyclophosphamide	17	no	3/3
				1 patient: prednisone	15	no	
			1/3 dexamethason	1 patient: dexamethason and methotrexate	9	no	
Biotti et al. [11]	1	-	prednisone	prednisone and azathioprine	7	no	unknown

* in a 2^nd^ described case, biopsy was initiated after failed initial prednisone therapy. PA results showed a low grade astrocytoma.

** this case report described a case in which biopsy was initiated after failed initial prednisone therapy. PA results showed a primary central nervous system lymphoma.

*** decline when maintenance treatment was tried to stopped.

All patients described so far (Table 2) were treated with intravenous and subsequent oral corticosteroids with gradually improvement of the clinical condition. Unfortunately, almost all patients relapsed following glucocorticoid tapering and required maintenance glucocorticoid or other immunosuppressive therapy. We present this new case of CLIPPERS to underline the importance of introducing maintenance immunosuppressive therapy after glucocorticoid treatment and the need to closely monitor for side effects during this prolonged therapy.

## Case presentation

A 63-year-old male presented with a five week history of double vision, rotational vertigo with tinnitus, progressive gait imbalance and apathy. One month earlier he had undergone tibia osteotomy surgery because of varus deformity. He used no medication. He had a history of alcohol abuse and did not smoke. He had no history of international traveling. There was no family history of neurological disease.

Except from postsurgical findings at his right knee general physical examination was normal. The blood pressure was 130/70 mmHg, pulse rate 78/minute, and he was afebrile. On initial neurological examination he was alert and cognitive functions were normal. His speech was dysarthric, chantering. Examination of the cranial nerves revealed a right abducens nerve paresis and an upbeat nystagmus. The pinprick sensation in the V3 distribution of the trigeminal nerve was symmetrically decreased. Other cranial nerve functions were normal. Strength was normal in muscles of the arms and legs and sensation was preserved. Vibration sense on both legs was diminished. The arms and legs on both sides showed atactic, dysmetric movements. Tendon reflexes were normal and the plantar reflexes were normal. He had an impressive gait ataxia and he was unable to walk without aid. His gait was further impaired due to the recent surgery.

As he was known with alcohol abuse, Wernicke’s syndrome was firstly assumed and he was promptly treated with intravenous thiamine.

However, the neurological condition of the patient further deteriorated. He developed difficulties swallowing and became incontinent for urine. His gait ataxia worsened and a slight spastic paraparesis developed.

In this patient with progressive cranial nerve dysfunction with ocular signs, ataxia and pyramidal tract signs, brain stem dysfunction with a variety of causes should be considered. The broad differential diagnosis included CNS lymphoma, primary CNS vasculitis, demyelinating diseases, paraneoplastic syndromes, sarcoidosis, tuberculosis and neurolues. The diagnosis of Wernicke’s syndrome was rejected as the clinical picture deteriorated and the pre-treatment thiamine level showed to be 118 nmol/L (normal range: 60–200 nmol/L).

**Figure 1:**
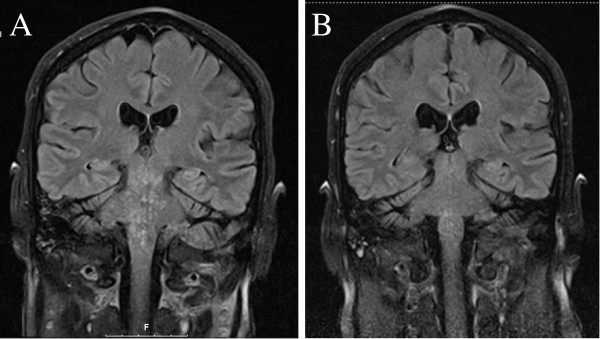
**Coronal FLAIR MRI images. A.** demonstrates the characteristic punctuate hyperintens lesions of the pons. **B.** shows diminishing of the pons-hyperintensity after glucocorticoid administration. The lesions were enhancing after gadolinium administration on T1 weighted images (not shown).

MRI T2-weighted and fluid-attenuated inversion recovery (FLAIR) images of the brain demonstrated distinct hyperintensity abnormalities consisting of punctuate lesions in the pons, brachium pontis and cerebellum (Figure 1A). The lesions were gadolinium enhancing but had no significant mass effect.

Blood levels of C-reactive protein, ESR, Vit B12 and Angiotensine Converting Enzyme levels were normal. Liver parameters were normal except for a slightly elevated gamma-GT of 65 U/L (reference <55 U/L). Thyroid functions were normal.

Antinuclear antibodies, double-stranded DNA antibodies, extractable nuclear antigens, antineutrophil cytoplasmic antibodies and antineuronal antibodies (anti-Hu, anti-Yo, anti-Ri, anti-Tr, anti-amphiphysine, anti-CV2 and anti-Ma2) were not detected.

Serologic tests on Syphilis, HIV, Borrelia Burgdorferi, anti-DNase B and anti-streptolysine titer were negative. Auramine staining and PCR for M.tuberculosis complex DNA were negative.

Analysis of the cerebrospinal fluid showed a raised protein level (protein 1.3 g/L, reference range 0.2–0.5) and a slight pleiocytosis (6,0 E06, reference <5 E06). Red cell count and glucose levels were normal. The IgG index was marginally increased (0.62, reference range 0.3–0.6), no oligoclonal bands were found. Cytology study reports showed normal amounts of lymphocytes, granulocytes monocytes and erythrocytes. Malignant cells were not detected.

Cultures of urine, blood and cerebral spine fluid were all negative.

MRI of the spinal cord and CT scanning of the chest, abdomen and pelvis showed no abnormalities. Total body PET CT scanning was negative for malignancies.

On the base of the typical MRI findings and exclusion of other possible diagnoses the diagnosis of CLIPPERS was presumed. The patient was treated with 1000 mg intravenous methylprednisolone daily for a period of three days, followed by oral prednisolone 80 mg per day. Within a week from starting treatment the symptoms gradually improved and on MRI two weeks from starting treatment the pontine lesions decreased in number and intensity (Figure 1B).

Maintenance immunosupressive therapy by oral prednisolone 80 mg per day was continued, adding methotrexate 2.5 mg weekly to the regimen as long-lasting immunosuppressive therapy was probably needed.

However, the therapy was complicated in several ways. Firstly, by the end of the first month of therapy our patient developed diabetes mellitus and deep venous thrombosis. Subsequently, severe liver function abnormalities developed after methotrexate was started (Table 3). Therefore, this drug was substituted by azathioprine in a step up dosage regimen up to 100 mg daily. Hereafter, a severe and long-lasting pancytopenia developed (Table 3). Bone marrow analysis showed hyperplastic erythropoiesis and hypoplastic granulopoiesis compatible with a side effect of the immunosuppressive therapy. The azathioprine was therefore stopped and prednisone 20 mg per day was continued.

**Table 3:** Laboratory results before and after treatment with immunosupressive therapy

**Liver function results on admission**		**Liver function results after treatment with methotrexate**	**Reference range**
ASAT	20 U/l	97 U/l	< 35
ALAT	18 U/l	172 U/l	< 45
Alkaline phosphatase	105 U/l	82 U/l	< 120
Bilirubin	9 umol/l	14 umol/l	<17
Gamma GT	65 U/l	394 U/l	< 55
**Hematologic results on admission**		**Hematologic results after treatment with azathioprine**	**Reference range**
Leucocytes	7,9 E09/l	1,5 E09/l	4,0–10,0
Erythrocytes	4,54 E12/l	1,96 E12/l	4,50–5,50
Hemaglobin	8,6 mmol/l	3,9 mmol/l	8,5–11,0
Hematocrit	0,42 l/l	0,18 l/l	0,40–0,50
Thrombocytes	229 E09/l	9 E09/l	150–400

After recovering from the pancytopenia, mycofenolaatmofetil was added starting at 500 mg daily and in increased dosage to 1000 mg bid. During this whole episode prednisone treatment was maintained at a dosage of 20 mg daily. A severe intercurrent herpes infection was treated with intravenous valcyclovir.

After a course of 18 months, our patient has not had any relapse of his neurological deficits. There were no cranial nerve deficits, no pareses and his gait was normal.

### Discussion

We made the diagnosis of CLIPPERS combining the clinical and typical MRI findings only after exclusion of other possible diagnoses. We choose not to perform a biopsy because of the localization of the abnormalities and because the imaging results and clinical presentation were very similar to the patients described by Pittock et al. [1].

In most of previously published studies it was mentioned that discontinuation of steroid treatment led to a clinical relapse (Table 2). Mean follow up time of all published reports so far was 28 months (3–144 months), and no successful steroid discontinuation was reported.

It is known that induction of remission by using glucocorticosteroids followed by maintenance glucocorticosteroid combining other immunosuppressive therapy in autoimmune diseases such as vasculitides should be continued for at least 2–5 years [12,13]. Since CLIPPERS has comparable pathologic features as in vasculitides consisting of perivascular lymphocytic infiltrate, it seems likely that tapering of maintenance therapy should be proposed only after 2–5 years of treatment guided by clinical monitoring and follow up MRI.

Finally we emphasize that in clinical practice one must pay attention to the adverse events, as described in our case. We recommend tapering immunosuppressive therapy in conjunction with frequent clinical monitoring and imaging, thus enabling us to treat any further relapse on time. Our findings support the need for further follow up studies to determine the duration of treatment.

## Conclusion

CLIPPERS presents with distinctive clinical and MRI-findings and can be diagnosed after exclusion other differential diagnoses [1,3]. Our patient confirmed the appropriateness of the initiation of treatment without histopathological confirmation.

A favourable clinical response to corticoid treatment lends further support to the diagnosis of CLIPPERS. We stress that careful clinical and laboratory monitoring is warranted because of the side effects of longstanding glucocorticosteroid and additional immunosuppressive therapy. Follow-up studies to determine the duration of treatment are necessary.

### Consent

Written informed consent was obtained from the patient for publication of this Case report and any accompanying images. A copy of the written consent is available for review by the Series Editor of this journal.

## Abbreviations

CLIPPERS: Chronic lymphocytic inflammation with pontine perivascular enhancement responsive to steroids; CNS: Central nervous system; CSF: Cerebrospinal fluid; FLAIR: Fluid attenuation inversion recovery; IV: Intravenous.

## Competing interests

The author(s) declare that they have no competing interests.

## Authors’ contributions

JW, AMMV, EJW, GBK and CEHS were responsible for manuscript: A. Writing of the first draft: JW and AMMV. B. Review and critique: EJW, GBK and CEHS. All authors have read and approved the final manuscript.

## Pre-publication history

The pre-publication history for this paper can be accessed here:

http://www.biomedcentral.com/1471-2377/13/49/prepub

## References

[B1] Pittock S Debruyne J Krecke K Giannini C van den Ameele J De Herdt V Chronic lymphocytic inflammation with pontine perivascular enhancement responsive to steroids (CLIPPERS) Brain 2010 133 2626 2634 10.1093/brain/awq164 20639547

[B2] Simon N Paratt J Barnett M Buckland M Gupta R Hayes M Expanding the clinical, radiological and neuropathological phenotype of chronic lymphocytic inflammation with pontine perivascular enhancement responsive to steroids (CLIPPERS) J Neurol Neurosurg Psychiatry 2011 83 15 22 22056964 10.1136/jnnp-2011-301054

[B3] Taieb G Waconge A Renard D Figarella-Branger D Castelnovo G Labauge P A new case of chronic lymphocytic inflammation with pontine perivascular enhancement responsive to steroids with initial normal magnetic resonance imaging Brain 2011 134 pt 8 e 182 author reply e183 10.1093/brain/awq390 21303857

[B4] Jones J Dean A Antoun N Scoffings D Burnet N Coles A Radiologically compatible CLIPPERS' may conceal a number of pathologies Brain 2011 134 pt 8 e 187 10.1093/brain/awr134 21653537

[B5] Sempere A Mola S Martin-Medina P Bernabeu A Khabbaz E Lopez-Celada S Response to Immunotherapy in CLIPPERS: Clinical, MRI, and MRS Follow-Up J Neuroimaging10.1111/j.1552-6569.2011.00631.x 21848680

[B6] Duprez T Sindic C Contrast-enhanced magnetic resonance imaging and perfusion-weighted imaging for monitoring features in severe CLIPPERS Brain 2011 134 pt 8 e184 author reply e186 21385752 10.1093/brain/awr034

[B7] List J Lesemann A Wiener E Walter G Hopmann D Schreiber S A new case of chronic lymphocytic inflammation with pontine perivascular enhancement responsive to steroids Brain 2011 134 pt 8 e185 author reply e186 21389007 10.1093/brain/awr035

[B8] Limousin N Praline J Motica O Cottier J Rousselot-Denis C Mokharti K Brain biopsy is required in steroid-resistant patients with chronic lymphocytic inflammation with pontine perivascular enhancement responsive to steroids (CLIPPERS) J Neurooncol 2012 107 1 223 224 10.1007/s11060-011-0724-0 21968945

[B9] Gabilondo I Saiz A Graus F Villoslda P Response to immunotherapy in CLIPPERS syndrome J Neurol 2011 258 2090 2092 10.1007/s00415-011-6068-z 21553269

[B10] Kastrup O van de Nes J Gasser T Keyvani K Three cases of CLIPPERS: a serial clinical, laboratory and MRI follow-up study J Neurol 2011 258 2140 2146 10.1007/s00415-011-6071-4 21556878

[B11] Biotti D Deschamps R Shotar E Maillart E Obadia M Mari I CLIPPERS: chronic lymphocytic inflammation with pontine perivascular enhancement responsive to steroids Pract Neurol 2011 83 2 3 10.1136/practneurol-2011-000043 22100944

[B12] Lapraik C Watts R Bacon P Carruthers D Chakravarty K D'Cruz D BSR and BHPR guidelines for the management of adults with ANCA associated vasculitis Rheumatology 2007 46 1615 1616 10.1093/rheumatology/kem146a 17804455

[B13] Ntatsaki E Mooney J Watts R ANCA vasculitis: time for a change in treatment paradigm? Not yet Rheumatology 2011 50 1019 1024 10.1093/rheumatology/ker002 21292735

